# Current News

**Published:** 1890-03

**Authors:** 


### Current News.

### CHICAGO AND VICINITY.

Professor Truman W. Brophy has returned from the Pacific Slope very much improved in health.

The annual meeting of the Chicago Dental Society was held on February 4. The entire day was devoted to scientific work,—the forenoon to clinics, the afternoon to the reading of papers and discussions; and the evening to a banquet at the Leland Hotel. Between fifty and sixty were present, including the guests of the society. A *marked* and *unmarked* feature of the banquet was the absence of all forms of intoxicating liquors.

La Grippe has seemed to have a special spite against the teachers in our medical and dental schools, and many of them have been obliged to find substitutes to fill their places for longer or shorter periods. Five of the professors in one of the dental colleges were laid off at the same time.

The annual dinner of the Chicago Dental Club occurred on the evening of February 24, at the Tremont House, at which Dr. W. X. Sudduth, of the International Dental Journal, Dr. F. H. Berry, of Milwaukee, Dr. C. R. E. Koch, of the Illinois State Board of Dental Examiners, and Dr. P. J. Kester, president of the Chicago Dental Society, were the honored guests.

After the dinner Dr. Sudduth delivered a lecture on "Cellular Morphology," illustrated by numerous photo-micrographs. He was assisted by Dr. L. D. McIntosh, of Chicago, who furnished for the occasion one of his new and improved lanterns.

Dr. Berry, of Milwaukee, demonstrated the "Construction of Appliances for Cleft Palate by a New and Improved Method." Forty-five were present at the dinner.

The Chicago Dental Society and the Chicago Dental Club inaugurated this year a new departure in the line of banquets. Instead of ordering an expensive dinner, at a late hour of the night, the societies sit down to the regular six o'clock dinner, served in a private room. This gives plenty of time for speech-making or literary and scientific work without robbing the next day, and saves a large item in expense, which can be spent to better advantage in other directions.

The World's Fair? Why, of course. Where should it go but to Chicago? What other city could make a success of it in such a limited space of time? This is what we hear on the street everywhere. Come and see, in 1892, if Chicago does not fulfil her promise.

---

At a mass meeting of over one hundred dentists, gathered from various parts of the United States, held in the city of New York, January 16, 1890, of which Dr. O. E. Hill was chairman, it was, on motion, unanimously

*Resolved*, That we thoroughly endorse the Dental Protective Association of the United States, and urge upon every member of the dental profession to join the association, and send to Dr. J. N. Crouse, of Chicago, its president, the initiation fee of ten dollars.

New York, January 16, 1890.

---

Call for an International Dental Congress in 1892.—At the Nineteenth Semi-annual Meeting of the New Jersey State Dental Society, held at the office of Dr. S. C. G. Watkins, Montclair, N. J., Saturday, January 11, 1890, the following resolution was passed:

"Deeming it fitting, and the proper time for holding an International Dental Congress in the year 1892, the New Jersey State Dental Society has appointed a committee to act in co-operation with like committees from all other dental societies throughout the United States. They would request your society to appoint a committee to meet with them at the Hoffman House, New York, on Tuesday afternoon, April 8, to formulate plans for the holding of the First International Dental Congress."

Trusting that this will meet with the approval of all Dental Societies and that your executive committees will appoint delegates at once.

Yours very respectfully,

S. C. G. Watkins, *President.*
Geo. Emery Adams, *Vice-President.*
Charles A. Meeker, *Secretary.*
Geo. C. Brown, *Treasurer.*

Fred A. Levy, Oscar Adelberg,
A. R. Eaton, B. F. Luckey,
G. Carleton Brown, C. F. W. Holbrook,
James G. Palmer, E. M. Beesley,
C. S. Stockton, Henry A. Hull,
Worthington Pinney.

